# Light quality regulates growth and flavonoid content in a widespread forest understorey medicinal species *Scutellaria Baicalensis* Georgi

**DOI:** 10.3389/fpls.2024.1488649

**Published:** 2024-12-16

**Authors:** Jingran Ma, Jiaxing Zhang, Lulu Xie, Ji Ye, Li Zhou, Dapao Yu, Qing-Wei Wang

**Affiliations:** ^1^ CAS Key Laboratory of Forest Ecology and Silviculture, Institute of Applied Ecology, Chinese Academy of Sciences, Shenyang, China; ^2^ University of Chinese Academy of Sciences, Beijing, China; ^3^ Changbaishan Xipo National Field Observation and Research Station for Forest Ecosystem, Baishan, China

**Keywords:** blue light, flavonoid biosynthesis, light-emitting diodes (LED), secondary metabolism, medicinal plants

## Abstract

**Introduction:**

Introduction: Light is not only essential for plant photosynthesis and growth, but also acts as a signal to regulate its secondary metabolism. Despite the influence of light quality on the yield and flavonoid compounds in commercial crops is well-documented, its role in regulating wild understorey species, particularly medicine plants whose flavonoid biosynthesis driven by multiple spectral regions of canopy sunlight, is less understood.

**Methods:**

To address it, we conducted a light-quality manipulation experiment on *Scutellaria baicalensis* Georgi, a widespread understorey medicinal species, with light-emitting diodes (LED). This study included eight treatments: UV-A (UV-A radiation), CK (control group), Green (monochromatic green light), and different combinations of blue and red light (R0B4: monochromatic blue light; R1B3: 25% Red+75% Blue light; R1B1: 50% Red+50% Blue light; R3B1: 75% Red+25% Blue light; R4B0: monochromatic red light).

**Results:**

Our results showed that light quality significantly drove morphology, biomass accumulation, and flavonoids biosynthesis in *S. baicalensis*. R0B4 treatment promoted growth and flavonoids accumulation, including baicalin, and wogonoside concentrations. In contrast, UV-A radiation and green light negatively impacted these parameters compared to CK treatment. Interestingly, plant biomass and flavonoid concentrations were lower in R1B3, R1B1 and R3B1 treatments compared to monochromatic blue or red light.

**Discussion:**

Our study found that red light may antagonize blue light-stimulated growth and flavonoids accumulation, indicating a complex crosstalk between photoreceptors. These findings highlight the importance of blue light for optimizing the yield and quality of *S. baicalensis* in the understorey cultivation. It provides practice suggestion for the efficient management and sustainable cultivation of understorey medicinal plants.

## 1 Introduction

Forests play a crucial role in maintaining ecosystems biodiversity and providing valuable medicinal resources (Deng et al., 2023; Borthakur et al., 2022). The forest understorey, a unique zone beneath the canopy, offers ideal conditions for cultivation and utilization of medicinal plants (Brelsford et al., 2022). To date, the demand for medicinal plants has been growing. However, due to a lack of theoretical knowledge and technological advancements in managements of understorey medical plants, suitable cultivation models that ensure both high yield and quality are scarce (Zhang et al., 2022; Zhu et al., 2022). Consequently, it is essential to comprehend the environmental factors that control the growth and production of medicinal compounds in understorey plants. This knowledge can provide a theoretical foundation for developing efficient and sustainable cultivation practices.

Medicinal compounds, primarily secondary metabolites, such as flavonoids, are influenced by numerous environmental factors, with light being especially important (Takshak and Agrawal, 2019; Ferreyra et al., 2021; Yan et al., 2023). Light is not only utilized for growth, but also serves as a cue mediating the production of secondary metabolites across its spectral range, from ultraviolet (UV)-B to red light (280–700 nm) (Wang et al., 2020; Zhang et al., 2022). Blue (400-500 nm) and red (600-700 nm) light are particularly effective in promoting plant biomass and secondary metabolites accumulation (Hogewoning et al., 2010; Neugart et al., 2021). In contrast, green light (500-600 nm) often acts as a shading light cue, generally counteracting the effects of blue light (Smith et al., 2017). UV-A radiation (315-400 nm) influences the synthesis of flavonoids and other secondary metabolites through photoreceptors such as phototropins and cryptochromes, although intense exposure can damage photosystem II (Chen et al., 2019; Lee et al., 2019; Han et al., 2022). Recent studies report that plant growth is strongly affected by the combination of blue and red lights (Hernández and Kubota, 2016; Miao et al., 2019). For example, optimal blue light to red ratios significantly enhances chlorophyll content, photosynthetic rates, and flavonoid production in basil (Lin et al., 2021). Additionally, the exclusion of blue light has been shown to reduce growth and biomass in pepper, cucumber and Tomato plants (Hogewoning et al., 2010; Kaiser et al., 2019; Li et al., 2020). These findings indicate that the impact of light quality on the yield and quality in commercial plant species, such as vegetables, are well documented. However, the impact of light quality on wild understorey species, especially medicinal plants whose flavonoid biosynthesis is primarily driven by habitat light conditions, remains less understood.

In forest ecosystems, sunlight is the most dramatic environmental factor due to the heterogeneous nature of canopy architecture (De Pauw et al., 2022; Nunes et al., 2022). Variations in light intensity and spectral composition arise from absorption, reflection, and shading by the canopy (Durand et al., 2021). These fluctuations can affect the growth of understorey plants in either a positive or negative way (Ma et al., 2024). Recent efforts to improve yield and quality of understorey medical plants have focused on modifying cultivation models, such as adjusting stand density, forest type, and canopy closure (Ruan et al., 2018). However, these models often overlook the interactive effects of different light qualities, and their regulatory mechanisms remain poorly understood. Researches in molecular biology and horticulture has shown that the combinations of blue, green and red light enhance photosynthesis and growth in cucumbers (Hogewoning et al., 2010; Hernández and Kubota, 2016), while blue light boosts flavonoid synthesis in *Arabidopsis*, regulated similarly to UV-A radiation (Rai et al., 2019). However, these findings do not account for the evolutionary genetic differences between understorey medicinal plants and model plants, making them difficult to apply in field cultivation. Therefore, it is crucial to study how the complex interactions of different light qualities influence plant growth, physiological and biochemical processes, especially the accumulation of secondary metabolites in understory medicinal plants.

This study aimed to examines how light quality affected the yield and quality of medicinal plants, *Scutellaria baicalensis* Georgi, commonly known as “Huang Qin” in Chinese, is a widely distributed medicinal plant in China, cultivated extensively in forest understorey and greenhouses (Do et al., 2021; Xiang et al., 2022). We utilized light-emitting diodes (LED) to provide different light treatments, including UV-A radiation, green, blue and red light, along with combinations of blue and red light at different ratios and focused on plant biomass, morphology, and the concentration of key bioactive components. *S. baicalensis* is well-known for its flavonoids, such as baicalin, wogonoside, baicalein, and wogonin, which serve as important quality indicators (Sun et al., 2020; Miao et al., 2022; Zhang et al., 2022). Recently such compound demand has been increasing, due to the potential function of *S. baicalensis* extracts and baicalin in treating coronavirus and SARS-CoV-2 (Liu et al., 2021; Hengphasatporn et al., 2022). Although previous studies have demonstrated that light intensity influences the accumulation of flavonoids in *S. baicalensis* (Fang et al., 2023), how light qualities regulate its growth and flavonoid biosynthesis remains poorly understood. Therefore, we formulated hypotheses: Blue light is the optimal spectral region for enhancing both yield and quality of *S. baicalensis* due to its essential role in regulating plant growth and flavonoids accumulation. On the other hand, such stimulation may be antagonized by red light due to the crosstalk between photoreceptors that share common light cues.

## 2 Materials and methods

### 2.1 Plant materials and seedling preparation

Our study was carried out in plant growth chamber at the Institute of Applied Ecology, Chinese Academy of Sciences, China (41.90°N, 123.61°E) from April to August 2022. Four cultivation frames, each consisting of three layers, were separated into eight distinct units. We used shading cloths to cover each unit, preventing light interference among the treatments. (**Supplementary Figure S1**). LED lamps (120 × 5 cm; Chengyue Equipment Co., Ltd, Shenzhen, China) were installed 30 cm above each plant to provide light. The lamp positions were adjusted as the plants grew to maintain consistent light intensity throughout the experiment.

The *S. baicalensis* seeds, sourced from Shenyang Agricultural University, China, were sterilized and germinated in pots with a volume of 307 cm³. After sprouting, they were cultured under full-spectrum LED lamps. We transplanted seedlings of similar height (approximately 10 cm) into various light treatments after the full expansion of their two true leaves. A total of 256 seedlings were used, with 32 assigned to each light treatment, and 4 seedlings used for flavonoid metabolism analysis. To ensure consistent light conditions, the position of pots was randomly exchanged twice a week. The plants were fertilized with a nutrient solution (N: P: K = 6%: 10%: 6%, HYPONEX Corporation, Japan) every two weeks.

### 2.2 Design of light spectra experiment

**Figure 1:**
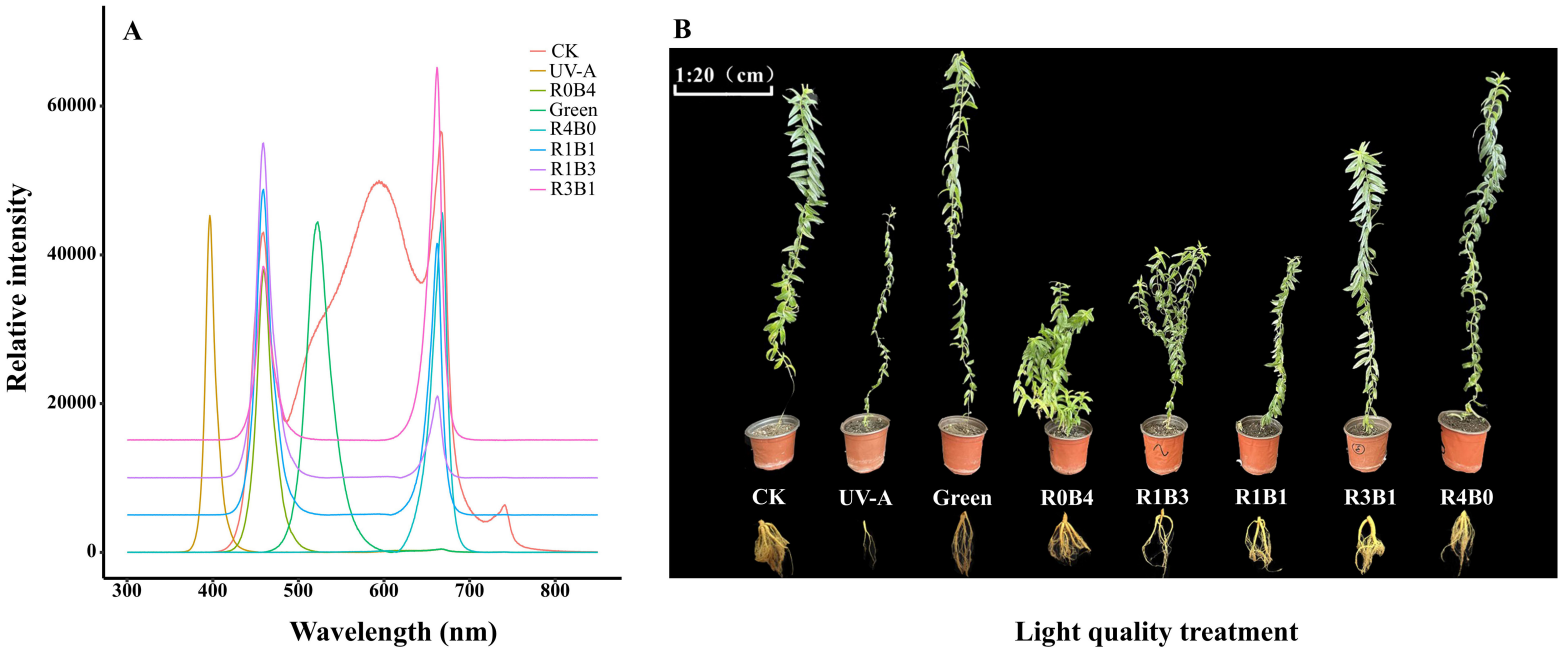
**(A)** Spectral compostion of light-emitting diodes (LEDs) used in the experiment. Spectra composition were determined using a spectroradiometer (Maya 2000 Pro, Ocean Optics Inc.). Spectra composition are shown for eight treatments, including CK (control, 100 μmol·m^-2^·s^-1^), UV-A (UV-A radiation, 10 μmol·m^-2^·s^-1^), Green (monochromatic green light, 100 μmol·m^-2^·s^-1^), R0B4 (monochromatic blue light, 100 μmol·m^-2^·s^-1^), R1B3 (25% Red+75% Blue light, 100 μmol·m^-2^·s^-1^), R1B1 (50% Red+50% Blue light, 100 μmol·m^-2^·s^-1^), R3B1 (75% Red+25% Blue light, 100 μmol·m^-2^·s^-1^), R4B0 (monochromatic red light, 100 μmol·m^-2^·s^-1^). To avoid overlapping spectral curves between different light treatments, we added 5000, 10000, and 15000 to the relative intensity values of the R1B1, R1B3, and R3B1 treatments, respectively. **(B)** Photographs of the growth of *Scutellaria Baicalensis* under different light quality treatments.

Based on the different spectral compositions of the LED lamps, the experiment included the following eight light treatments: CK (control, 100 μmol·m^-2^·s^-1^), UV-A (UV-A radiation, 400 nm, 10 μmol·m^-2^·s^-1^), Green (monochromatic green light, 520 nm, 100 μmol·m^-2^·s^-1^), R0B4 (monochromatic blue light, 460 nm, 100 μmol·m^-2^·s^-1^), R1B3 (25% Red+75% Blue light, 100 μmol·m^-2^·s^-1^), R1B1 (50% Red+50% Blue light, 100 μmol·m^-2^·s^-1^), R3B1 (75% Red+25% Blue light, 100 μmol·m^-2^·s^-1^), R4B0 (monochromatic red light, 660 nm, 100 μmol·m^-2^·s^-1^). The spectral composition of different light treatments (**Figure 1A**) was analyzed using a calibrated spectroradiometer (Maya 2000 Pro, Ocean Optics Inc). The light intensity from the LED was set at 100 ± 10 μmol m^-2^ s^-1^, measured with a quantum sensor (LI-190SA, LI-COR, USA) and recorded with a data-logger (LI-1500, LI-COR, USA). During the experiment, the plants were cultivated for 120 days under a 12-hour daily photoperiod. The growth chamber maintained an average temperature of 22 ± 2°C and relative humidity at 65 ± 5%.

### 2.3 Measurements of physiological and biochemical traits

In August 2022, prior to harvesting the seedlings for various analyses, we measured the maximum quantum yield of Photosystem II (*F*_v_/*F*_m_) using a chlorophyll fluorometer (MINI-PAM-II, Heinz-Walz GmbH, Germany). This measurement, taken after a 30-minute dark adaptation period to allow relaxation of the PSII reaction centers, reflects the peak photochemical efficiency of PSII. Additionally, we used rapid light curves to determine the effective quantum yield of PSII (YII), following the fitting equation from (Jakob et al., 2005), which quantifies the active chemical quantum yield of PSII under illuminated conditions. Concurrently, the chlorophyll content of leaves was determined non-destructively using a polyphenol meter (Model MPM-100, USA).

### 2.4 Measurements of morphology, biomass and growth rate

Morphological traits, including plant height, basal diameter, and root length, were measured using a straightedge and vernier calipers. Leaf thickness was determined using a Mitutoyo Thickness Gage (Japan). In August 2022, four *S. baicalensis* seedlings were sampled randomly from each light quality treatment. After harvesting, total leaf area was measured by scanning all leaves and analyzing the images with Fiji software (www.fiji.sc, ImageJ). Plant samples were dried at 65°C for 3 days in oven to quantify the biomass of leaf, stem, and root.

The relative growth rate was calculated using the formula:


$$\begin{array}{c} \text{Relative growth rate}\\ =\frac{\text{ln}\left(\text{Final biomass}\right)-\text{ln }\left(\text{Fnitial average biomass}\right)}{\text{Days between samplings}} \end{array}$$

This served as an index of the overall seedling growth status (Oguchi et al., 2017). The initial average biomass was derived from the mean biomass of 10 seedlings sampled prior to the light quality treatments. The root-to-shoot ratio (R/S) was calculated as:


$$\text{R}/\text{S}=\frac{\text{Belowground biomass}}{\text{Aboveground biomass}}$$

### 2.5 Extraction and analysis of total flavonoids concentrations

The total flavonoids concentration in different organs were determined using the method of ultraviolet spectrophotometer (Gao et al., 2016). Briefly, 10 mg of dried powdered samples was extracted with 5 mL of 50% ethanol and shaken for 45 s for thorough mixing. The ethanol-extracted samples were then placed in an ultrasonic meter at 60°C for 30 minutes. After ultrasonication, the samples were cooled and centrifuged at 3500 rpm for 15 minutes. The supernatant was poured into a 10 mL volumetric flask, diluted with 50% ethanol, and thoroughly mixed. This procedure was repeated twice, and the supernatants were pooled for flavonoid analysis. Finally, 300 μL of the supernatant was analyzed colorimetrically at 620 nm using a spectrophotometer (Thermo Fisher Scientific, Finland).

### 2.6 Extraction and analysis of flavonoids composition and concentrations by (high-performance liquid chromatography) HPLC

Frozen plant tissue samples were ground through a 100-mesh sieve. Then, we dissolved 20 mg of the powdered samples in methanol and homogenized for 45 seconds using a vortex mixer. The extraction was refrigerated at 4°C for 15 minutes, followed by an additional 30 seconds of homogenization. Subsequently, we centrifuged the mixture at 3500 rpm for 5 minutes. The supernatant was collected, and the precipitation process was repeated five times with methanol. The combined extracts were concentrated under a stream of nitrogen gas to remove the solvent, leaving a dry residue. This dry extract was then redissolved in a 1:1 mixture of methanol and water to a final volume of 1 mL, serving as the sample solution for flavonoid compound analysis.

The flavonoid compounds were analyzed using High-Performance Liquid Chromatography (HPLC) with a Waters e2695 separation module (Waters Corp., Milford, MA, USA). Chromatographic separation was performed on a Waters Symmetry C18 column (150 mm × 4.6 mm, 5 μm particle size) at a column temperature of 30°C. The mobile phase consisted of two solvents: W1 (0.5% formic acid aqueous solution) and W2 (acetonitrile). The gradient elution profile was as follows: 0-5 minutes, 20% W2; 5-30 minutes, linear gradient from 20% to 30% W2; 30-55 minutes, linear gradient from 30% to 55% W2; and 55-60 minutes, return to 20% W2. The flow rate was set at 0.8 mL/min, and the injection volume was 10 μL. Compounds were detected at 280 nm. Quantitative analysis was conducted using four reference compounds: Baicalin (C_21_H_18_O_11_), Baicalein (C_15_H_10_O_5_), Wogonoside (C_22_H_20_O_11_), and Wogonin (C_16_H_12_O_5_), all with a purity of ≥98% HPLC grade, obtained from Shanghai Yuan Ye Biological Technology Co., Ltd. and Shanghai Jin Sui Biological Technology Co., Ltd. A 10 μL aliquot of both reference and test solutions was subjected to the described chromatographic conditions. Peak times were identified, and peak areas were measured to determine the concentrations, presented as mg·g^-1^ DW (dry weight) of different organs of *S. baicalensis*.

### 2.7 Statistical analysis

We used a one-way ANOVA to examine the impact of light quality on seedling growth and flavonoid concentration using the *stats* package in R. Before performing statistical analyses, we tested the data for homogeneity and normality. We used Fisher’s Least Significant Difference (LSD) test to examine the differences among light quality treatments, using the *agricolae* package. The light quality influence index (LQI) provides a comprehensive measure of the impact of light quality on plant yield and quality. It is calculated using the following formula:


$$\text{LQI}=\frac{\text{Average value of light quality treatments}}{\text{Average value of control}\;\left(\text{CK}\right)\;\text{group}}$$

All statistical analyses were performed using R software (ver. 4.3.3, R Development Core Team, 2024).

## 3 Results

### 3.1 Response of plant growth and morphological characteristics to light quality

**Figure 2:**
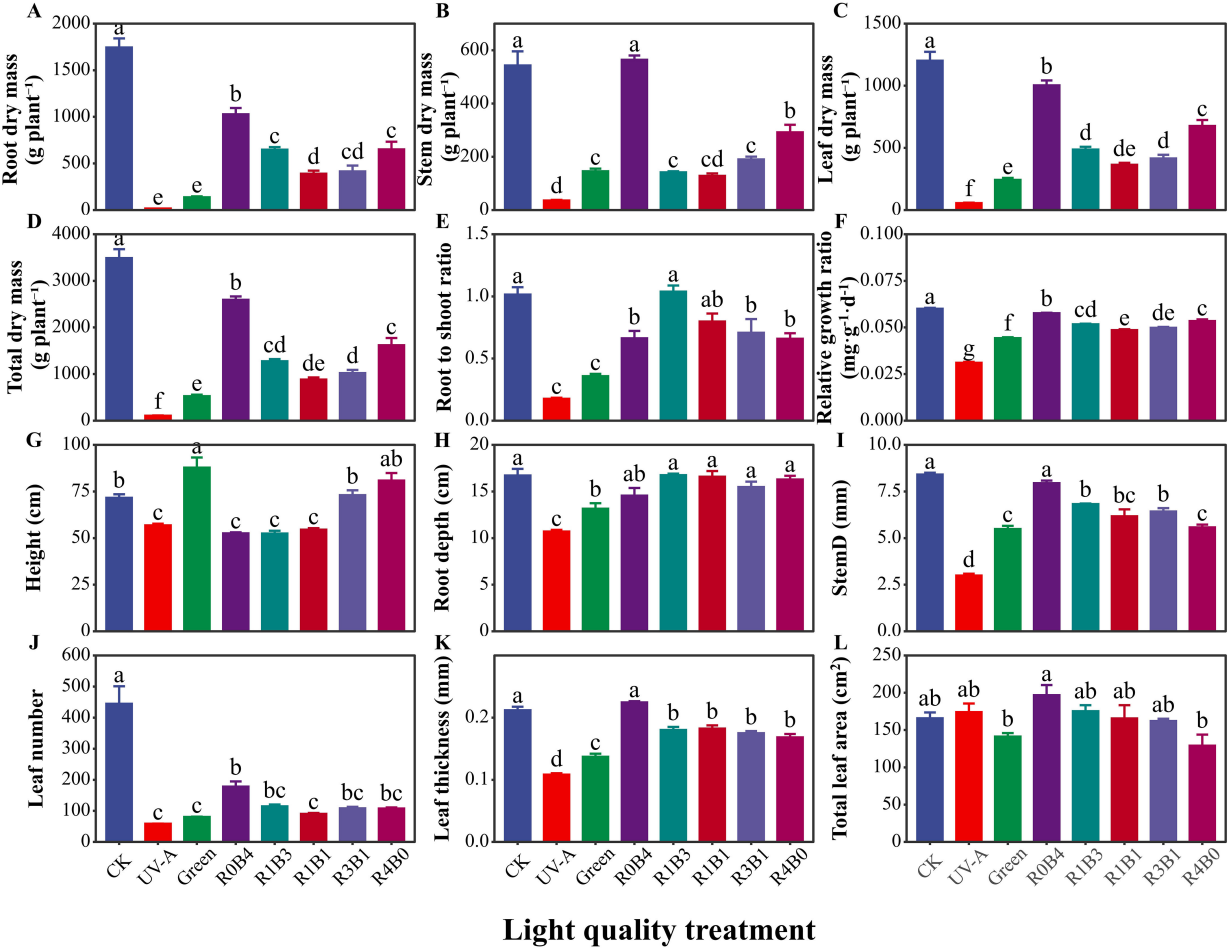
Response of plant growth and morphology to light quality in *Scutellaria Baicalensis*. **(A–F)** plant growth characteristics in each treatments, **(G–L)** plants morphlogical traits in each treatments. Abbreviations of light treatments are the same as in **Figure 1**. Different letters mean significant differences at *p* < 0.05 based on LSD test. Error bars represent the standard error (mean ± SE, n = 4).

The effects of various light quality treatments on the growth and morphology in *S. baicalensis* are showed in **Figure 1B**. Plants under UV-A treatment exhibited the lowest morphological and growth characteristics, including stem diameter, root depth, leaf number, leaf thickness, biomass, and relative growth rate (**Figure 2**). Compared with the CK group, green light treatment has positive effect on the decrease of total dry biomass and growth by 84.9% and 26.3%, respectively, except for UV-A treatment (**Figures 2A–E, G**). The plants under R0B4 exhibited highest leaf thickness and total leaf area (**Figures 2K, L**), with a pronounced increased in relative growth rate, root biomass, and total biomass, except for the CK group (**Figures 2D–F**). In contrast, R4B0 reduced root biomass and total biomass by 53.6% and 62.7%, respectively, compared to the CK group (**Figures 2A, D**). Likewise, R1B3, R1B1, and R3B1 treatments decreased whole plant and root biomass by 63.4% and 62.8%, 70.7% and 76.2%, and 74.7% and 77.6%, respectively, compared to the CK group (**Figures 2A, D**).

### 3.2 Response of physiological and biochemical traits to light quality

**Figure 3:**
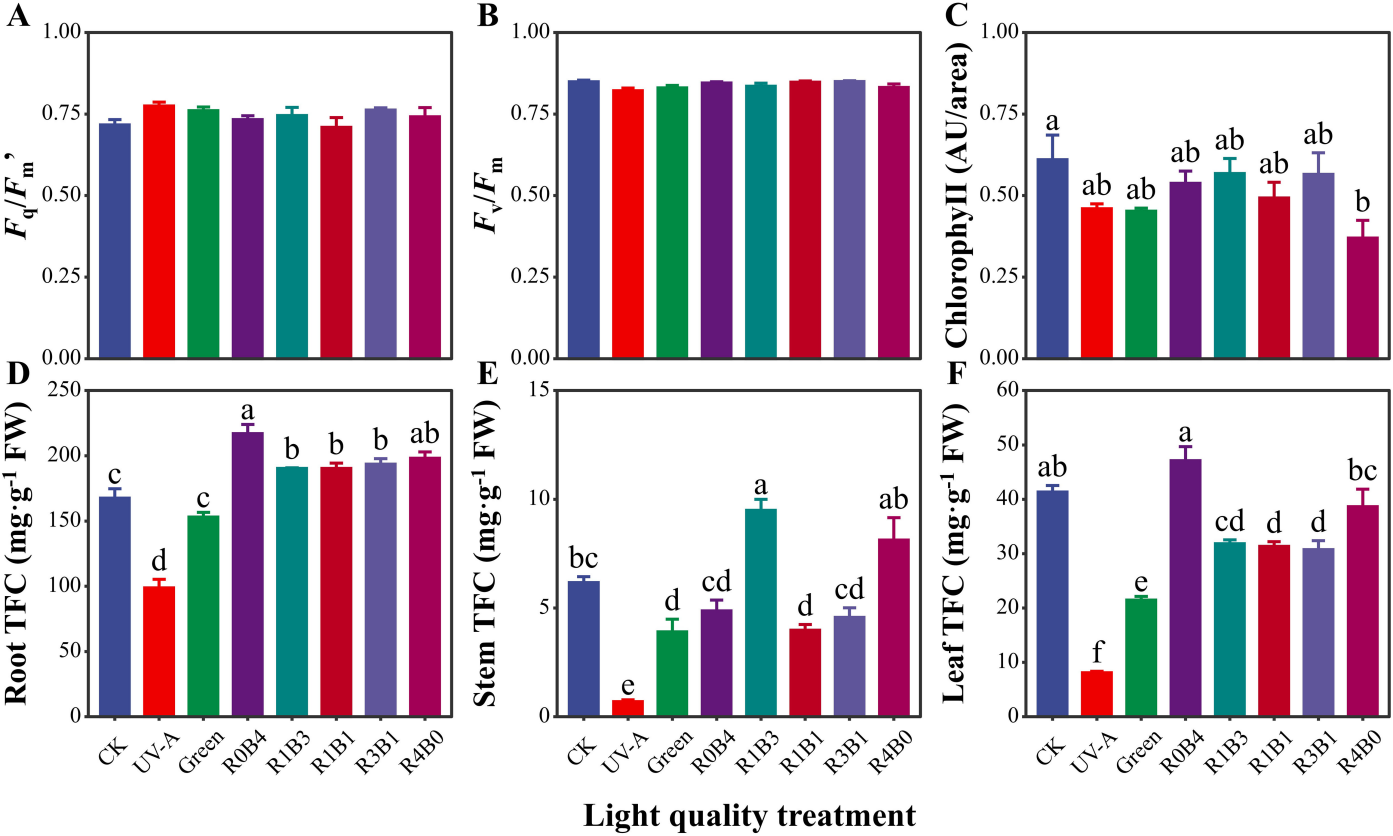
Response of physiological and biochemical traits to light quality in *Scutellaria Baicalensis*. **(A)** *F*_q_/*F*_m_’, leaf *F*_q_/*F*_m_’ or Φ_PSII_ measured under the ambient light condition, giving the effective quantum yield of photosystem II photochemistry, **(B)** *F*_v_/*F*_m_, leaf *F*_v_/*F*_m_ measured by MINI-PAM after dark acclimation, giving the maximum quantum yield of photosystem II photochemistry **(C)** Chlorophyll, leaf chlorophyll content measured by Dualex, **(D–F)** total flavonoids concentration (TFC) of each organ. Abbreviations of light treatments are the same as in **Figure 1**. Different letters mean significant differences at *p* < 0.05 based on LSD test. Error bars represent the standard error (mean ± SE, n = 4).

Values of *F*_q_/*Fm*’ and *F*_v_/*F*_m_ were similar across light quality treatments (**Figures 3A, B**). However, seedlings under R4B0 treatments exhibited the lowest chlorophyll content, while that in CK group had the highest value (**Figure 3C**). An interesting pattern emerged in the total flavonoid concentration (TFC): both root and leaf TFC were highest under R0B4, surpassing other treatments (**Figures 3D–F**). In contrast, TFC accumulation in roots and leaves showed a significantly reduction under the R1B3, R1B1 and R3B1 compared to R0B4 and R4B0 treatments (**Figures 3D, F**).

### 3.3 Response of flavonoid concentrations to light quality

**Figure 4:**
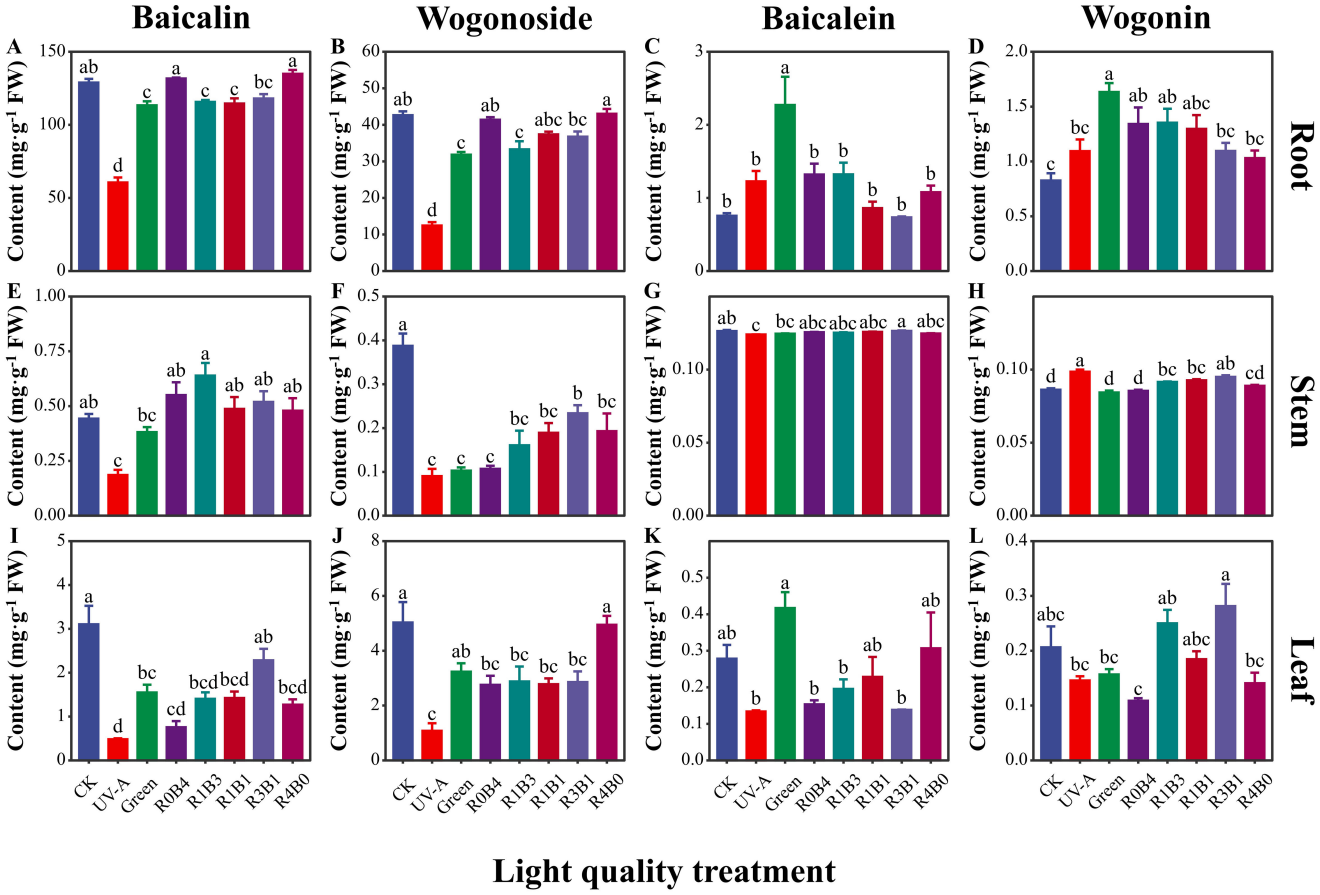
Response of four flavonoids to light quality in different organs of *Scutellaria Baicalensis*. **(A–D)** Four flavonoids (baicalin, wogonoside, baicalein and wogonin) in root; **(E–H)** four flavonoids in stem; **(I–L)** four flavonoids in stem. Abbreviations of light treatments are the same as in **Figure 1**. Different letters mean significant differences at *p* < 0.05 based on LSD test. The error bars represent the standard error (mean ± SE, n = 3).

The concentrations of different flavonoids components in different organs of *S. baicalensis* were quantified using HPLC, as shown in the chromatogram in **Supplementary Figure S2**. Among these flavonoids, baicalin concentration was highest than other flavonoids. Baicalin concentration was lower in the R1B3, R1B1, and R3B1 treatments compared to R0B4 and R4B0 treatments (**Figure 4A**). Similarly, wogonoside concentration was higher under CK, R0B4, and R4B0 treatments, following the same pattern as baicalin (**Figure 4B**). Furthermore, baicalin and wogonoside concentration were higher in R1B3, R1B1 and R3B1 treatments compared to R0B4 and R4B0 treatments in stem and leaf (**Figures 4E, F, I, J**). Baicalein concentration was consistently lower in all other treatments compared to the green light treatment, with the R3B1 treatment showing the lowest concentration at only 32.3% of that in the green light group (**Figures 4C, G, K**). The trend in wogonin concentration was similar with baicalein, with the highest levels recorded under green light, amounting to 198% of those in the CK group (**Figure 4D**). We observed higher wogonin concentration under R1B3, R1B1 and R3B1 treatments than R0B4 and R4B0 treatments (**Figures 4H, L**).

### 3.4 Light quality influence index

**Figure 5:**
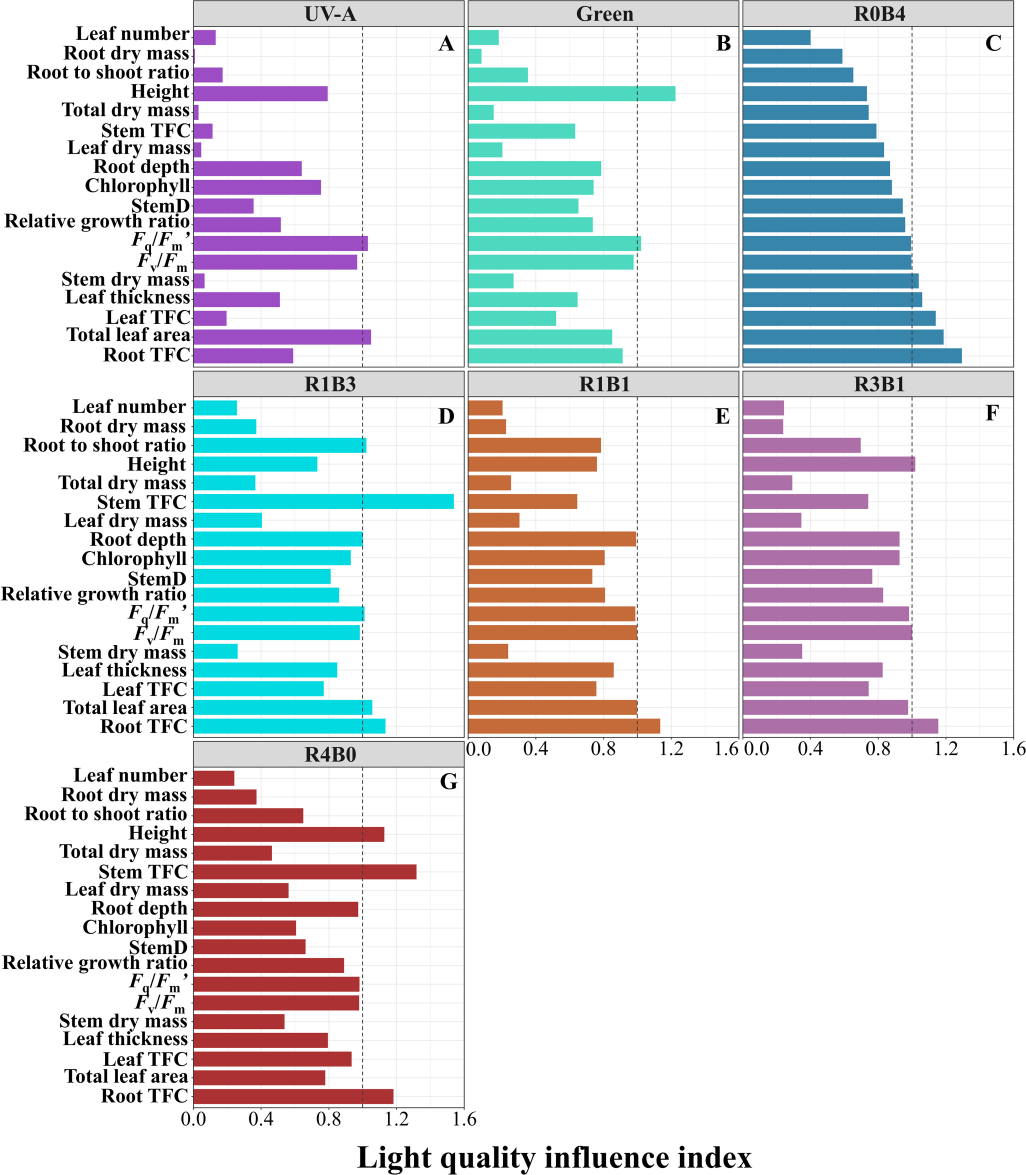
Light quality influence index (LQI) illustrating the effect of different light quality treatments on yield and quality in *Scutellaria Baicalensis*. **(A, B)** The LQI was calculated from UV-A and Green treatments, **(C–G)** different combinations of blue and red light treatments. The vertical dashed line in the figure at a horizontal coordinate of 1.0 is the average value of the traits of the CK group. Bars for each treatment are arranged by increasing mean values of LQI in R0B4 treatment. Abbreviations are the same as in **Figure 1**.

To obtain an integrated view of the yield and quality in *S. baicalensis*, we generated LQI to synthesize the functional profile of *S. baicalensis* in response to different light treatments (**Figure 5**). The UV-A groups exhibited lower LQI in dry mass, relative growth rate, and inferior physiological and biochemical traits compared to the CK group (**Figure 5A**). Green light treatment showed higher LQI in plant height than CK group (**Figure 5B**). Importantly, blue light treatment showed a relatively higher LQI in leaf and root flavonoid concentrations, total leaf area, leaf thickness, and *F*_v_/*F*_m_ compared to the CK group (**Figure 5C**). The R1B3 treatment displayed a similar LQI in root flavonoid concentrations to the R1B1 and R3B1 treatments but had a higher LQI in total leaf area compared to these groups (**Figures 5D–F**). The R4B0 treatment exhibited higher plant height and stem and root flavonoid concentrations compared to the CK group (**Figure 5G**). Monochromatic blue light more effective in enhancing the growth and functional components of *S. baicalensis*, combinations of blue and red light, proving to be more favorable for improving both yield and quality.

## 4 Discussion

### 4.1 Blue light is the most effective spectral region for promoting growth and flavonoids accumulation in *S. baicalensis*

As expected, the variation in morphology and growth indicates that blue light is the optimal spectral region for enhancing yields in *S. baicalensis*. Specifically, the R0B4 treatments exhibited higher plant height, total leaf area, total biomass, and relative growth rate compared to other light treatments, with the exception of the control group (**Figures 2**, **5**). Blue light promotes plant growth by stimulating photosynthesis and regulating the photomorphogenesis is well characterized (Košvancová-Zitová et al., 2009; Matthews et al., 2020), as mediated by photoreceptor protein kinase phototropins (Inoue and Kinoshita, 2017; Li et al., 2023). However, our results did not support a direct role of photosynthesis in this growth response (**Figures 3A, B**), indicating that photomorphogenesis is a more likely explanation. This is aligning with previous research that highlight the critical roles of blue light in mediating growth and morphology of plants (Zhang et al., 2019; Liang et al., 2021). Blue light notably enhanced the number of leaves (**Figures 2A–D, J**), likely due to its effects on branching and bud burst, which promote biomass accumulation (Girault et al., 2008; Li et al., 2020). Similarly, green and red light effectively enhanced plant height (**Figures 2G, H**), probably by stimulating cell elongation (Moon et al., 2006; Son and Oh, 2013).

Blue light also positively affected the total flavonoid concentration in the different organs of *S. baicalensis*, similar to its effects under full-spectrum light. This suggests that blue light is a key spectral region mediating the accumulation of flavonoid compounds, particularly baicalin and wogonoside, in *S. baicalensis*. Blue light is known to be crucial for the accumulation of secondary metabolites (Brelsford et al., 2022; Zhang et al., 2020), a process regulated by CRYs (Tissot and Ulm, 2020). For example, grapevine (*Vitis vinifera*) shows optimal flavonoid concentrations under high blue light conditions (Brelsford et al., 2022). In contrast, red light treatment has lower chlorophyll content compared to the CK group and other light treatments, likely due to a phenomenon known as ‘red light syndrome’ in *S. baicalensis* (Trouwborst et al., 2016). While red light does promote flavonoid production, it also seems to affect chloroplast positioning, potentially reducing photodamage (Miao et al., 2019).

UV-A radiation can perform similar functions as blue light by interacting with the same photoreceptors, such as CRYs (Rai et al., 2021; Zhang et al., 2020). Both UV and blue light have been shown to induce the biosynthesis of secondary metabolites, such as phenolic compounds and flavonoids (Banerjee and Batschauer, 2005; Brelsford et al., 2019; Palma et al., 2021). Combining UV-A radiation with visible light has been reported to boost antioxidant capacity and elevate flavonoid concentrations (Chen et al., 2019; Lee et al., 2019). However, in our study, UV-A radiation showed a negative effect on flavonoid and biomass accumulation compared to blue light. The photosynthetic capacity of *S. baicalensis* under UV-A treatment was comparable to other light treatments, suggesting that the observed reduction in flavonoid concentration may be influenced by phototropins 1 and 2, which affect hormone synthesis and gene expression (Banerjee and Batschauer, 2005; Brelsford et al., 2019). These findings are consistent with previous studies (Siipola et al., 2015; Zhang et al., 2020), confirming that blue light is more effective than UV-A radiation in promoting flavonoid biosynthesis.

### 4.2 Red light can antagonize blue light-stimulated growth and flavonoid accumulation in *S. baicalensis*

The interaction between different light qualities can be either synergistic or antagonistic due to the complex signaling networks involving various photoreceptors (Casal, 2013; Smith et al., 2017). For instance, green light can counteract the stomatal opening induced by blue light, mediated by uncharacterized green light photoreceptors and CRYs (Aasamaa and Aphalo, 2016; Smith et al., 2017). In our study, we observed lower biomass accumulation, relative growth rates and flavonoids concentration in the R1B3, R1B1, and R3B1 groups, compared to the R0B4 or R4B0 treatments (**Figures 2**, **5**). This indicates that red light may antagonize the growth and flavonoid accumulation stimulated by blue light, likely due to the photoreceptor’s crosstalk, as we hypothesized (Miao et al., 2019; Matthews et al., 2020). This finding is consistent with previous research, which suggests that red light can influence physiological processes traditionally thought to be blue light-dependent, through photoreceptor protein kinases such as phototropins (Matthews et al., 2020; He et al., 2023). For example, combining red and blue light has been observed to suppress stem extension more than either light alone, a phenomenon known as “coaction” (Casal et al., 1998; Folta and Spalding, 2001; Hernández and Kubota, 2016). Despite this, other studies have demonstrated that combinations of blue and red light can effectively enhance plant growth, potentially due to higher overall light intensity (Riikonen et al., 2016; Lin et al., 2021; Zhang et al., 2022). Notably, in our study, seedling growth was most inhibited with a 1:1 blue-to-red light ratio, suggesting that increasing the proportion of blue light could be more beneficial for field cultivation.

Our study also revealed that monochromatic blue and red light were more effective at enhancing flavonoid accumulation compared to the combinations of blue and red light. This contrasts with previous research showing that an optimal ratio of red and blue light can significantly increase cannabidiol levels in *Cannabis sativa* (Wei et al., 2021) and promote flavonoid accumulation in *Scutellaria baicalensis* (Zhang et al., 2022). The discrepancy may be due to the lower light intensity used in our experiments, where interactions between monochromatic red and blue light were more noticeable (Matthews et al., 2020). For instance, blue light can counteract the “red light syndrome,” a condition of reduced photosynthetic capacity caused by red light in cucumber plants (Miao et al., 2019). Our findings suggest that monochromatic blue light is particularly effective for stimulating the accumulation of key active compounds, such as baicalin and wogonoside, in *S. baicalensis*.

In this study, we employed LEDs as artificial light sources, which may not fully replicate the complexities of natural light environments. Consequently, the effects observed in our experiment might differ under natural habitat conditions. Furthermore, since this study focused on a single species, future research should include a range of medicinal plants to draw more comprehensive conclusions.

### 4.3 Implications for future understorey medical plants cultivation

The present study aimed to connect the yields and qualities of understorey medicinal plants to optimal light conditions. We propose that field cultivation practices can be optimized to enhance both growth and flavonoid production in medicinal plants. Specifically, increasing the proportion of blue light could effectively boost production in intensive cropping systems. Additionally, employing genetic breeding techniques to develop varieties adapted to the complex understorey light environment is advisable. Furthermore, implementing optimized light quality systems for cultivating medicinal plants could support commercial needs for efficient, large-scale, and precise management.

## 5 Conclusions

Our results demonstrated that blue light was the optimal spectral region to promote growth and flavonoid accumulation of *S. baicalensis*, leading to increased production of baicalin and wogonoside. In contrast, UV-A radiation and green light negatively affected both biomass and flavonoid accumulation. Interestingly, the combination of red and blue light proved to be less effective than using either monochromatic blue or red light alone, suggesting complex interactions between these light spectra. These findings offer new insights into how light qualities influences the yield and quality of understorey medicinal plants and provide valuable insights for optimizing cultivation practices and ensuring sustainable management in the traditional medicine industry.

## Data Availability

The original contributions presented in the study are included in the article/**Supplementary Material**, further inquiries can be directed to the corresponding author/s.
