# Supplementary material for: Light quality regulates growth and flavonoid content in a widespread forest understorey medicinal species Scutellaria Baicalensis Georgi
Source: Front Plant Sci. 2024 Dec 16;15:1488649. doi: 10.3389/fpls.2024.1488649 (PMC11683125; doi:10.3389/fpls.2024.1488649)
Supplement: Supplementary file 1 [file Table1.docx]

***Supplementary Material***

## Supplementary Figures


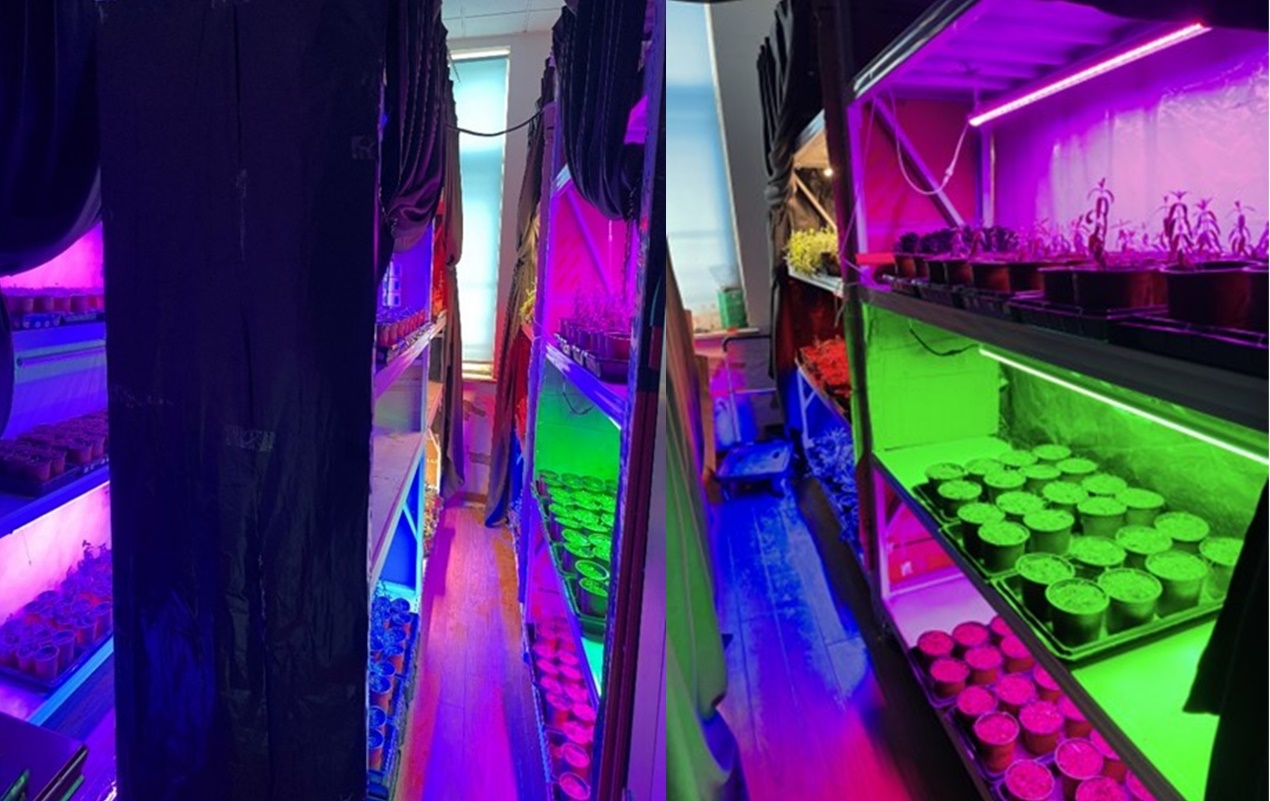


**Supplementary Figure 1. Photograph of the light quality treatments in different growth chambers.**


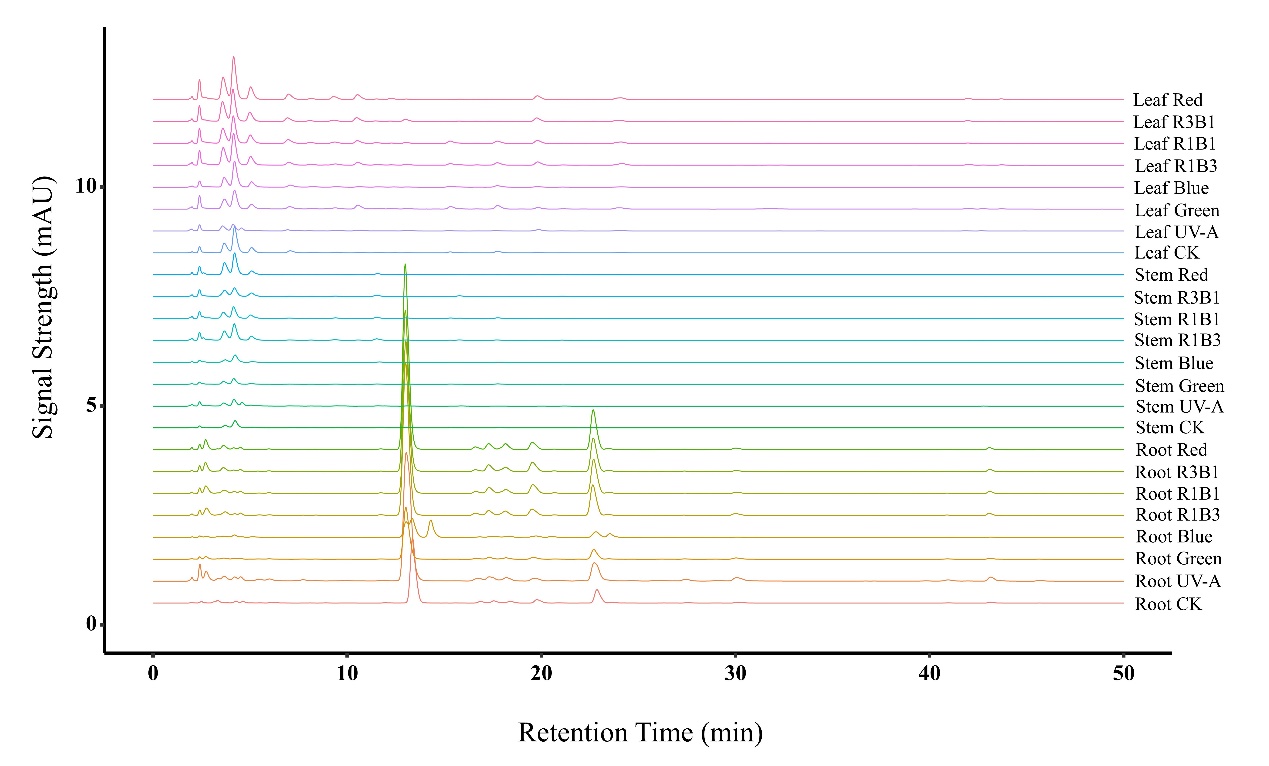


**Supplementary Figure 2. HPLC fingerprint of flavonoids in different plant organs of *Scutellaria baicalensis* under different light quality treatments.**

**
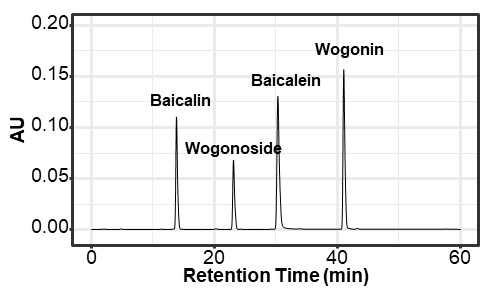
**

**Supplementary Figure 3. HPLC chromatograms (280 nm) of four flavonoids in *Scutellaria Baicalensis*.**
